# Tobacco Retailer Perspectives on Potential Phase Out of Tobacco Sales in South Australia: An Exploratory Survey

**DOI:** 10.1002/hpja.70257

**Published:** 2026-10-15

**Authors:** Joshua Trigg, Ella Vallelonga, Chelsea Falso, Ashleigh Guillaumier, Heewon Kang, Jennifer Baldock, Ryan Calabro, Janet Hoek, Coral Gartner

**Affiliations:** ^1^ College of Medicine and Public Health Flinders Health and Medical Research Institute, Flinders University Bedford Park Australia; ^2^ School of Public Health The University of Queensland Herston Australia; ^3^ Cancer Council South Australia Eastwood Australia; ^4^ Department of Public Health The University of Otago Wellington New Zealand

**Keywords:** policy, retailers, sales restriction, smoking, tobacco, tobacco control

## Abstract

**Issue Addressed:**

To examine tobacco retailers' perspectives on the potential phase out of tobacco sales in South Australia, a retail‐based tobacco endgame strategy that directly targets product availability.

**Methods:**

A cross‐sectional mixed‐methods survey of licensed tobacco retailers conducted in South Australia in April 2025. The survey included 70 licensed tobacco retailers and assessed anticipated impacts of a retail phase out, strategies they might adopt in response and the support they would seek. Both quantitative ratings and open‐text qualitative responses were analysed using descriptive statistics and inductive thematic analysis.

**Results:**

Retailers rated legal requirements, declining profitability and reduced consumer demand as the most important drivers for them to cease tobacco sales, while few highlighted public or community health considerations. One‐third reported needing lead‐in time to transition, citing financial compensation, business planning support and opportunities for product substitution as priorities. Concerns about illicit tobacco trade featured prominently, with many describing reduced legal sales, financial loss and security risks. Retailers held mixed views on phased transition to ending tobacco sales.

**Conclusions:**

Some retailers view tobacco as increasingly unprofitable, yet business sustainability concerns and illicit trade complicate retailer support for mandated phase out. Clear timelines and targeted transition support are critical to increasing retailer confidence and policy feasibility.

**So What?:**

By aligning tobacco retail phase‐out policies with retailer support, governments may be able to reduce tobacco availability while limiting impacts on retailers' livelihoods; further research is needed to confirm how this approach would affect policy acceptability and business transitions at scale.

## 1 Introduction

Tobacco smoking is the leading cause of preventable death in Australia, killing over 24 000 people each year, including more than 10 000 from cancer [1]. It accounts for 15% of deaths among those aged over 45 years and nearly one‐quarter of premature deaths among adults aged 45–75 years [1]. Yet tobacco remains widely available, with an estimated 40 000 retailers selling tobacco products nationwide [2].

Australia's National Tobacco Strategy (2023–2030) recognises tobacco use as the single greatest contributor to preventable mortality and health inequity impacts [3]. In South Australia, daily smoking prevalence has plateaued at around 8% of adults, with rates far higher among Aboriginal and Torres Strait Islander peoples (35% in 2022–23) [4, 5]. These figures underline the need for additional evidence‐based tobacco control approaches to meet smoking prevalence reduction targets. South Australia is currently leading Australian jurisdictions in efforts to lower tobacco smoking prevalence, reflecting its comparatively strong performance on national tobacco and vaping reform benchmarks [6]. However, phasing out retail tobacco sales, a measure that makes visible, permanent changes in retail tobacco product availability and exposure, is yet to be considered.

Phasing out tobacco sales is a central approach in efforts to achieve a tobacco endgame (i.e., strategies designed to permanently end the sale and use of tobacco products with a defined timeframe) [7]. A greater number of tobacco retailers is consistently associated with higher smoking prevalence and lower cessation rates, while lower availability is linked to less uptake and greater quit success [8, 9]. Modelling evidence suggests that dramatic reductions in retailer numbers of up to 95% may be required to effectively reduce smoking prevalence [10, 11]. Most importantly, there is high public support for a tobacco retail phase out, with 62% of Australians agreeing that ceasing tobacco sales would be a ‘good thing’ and 65% agreeing that retailers should ‘seriously be thinking about transitioning out of selling cigarettes’ [2].

Strategies focused on reducing tobacco sales have proven effective internationally [12]. Tobacco retail phase outs in Beverley Hills and Manhattan Beach were found to have limited negative financial repercussions for businesses when transitional methods were implemented to support retailers. Following the phase out, surrounding neighbourhoods did not incur a significant increase in tobacco sales, indicating the measure eliminated sales in local stores without causing major displacement to neighbouring areas [13]. Australia is a global leader in tobacco control, having enacted a suite of stronger measures that signal a shift towards a tobacco endgame, including novel health warnings, pack inserts and stronger penalties for illicit tobacco sales [14]. As tobacco retail phase out is gaining international momentum, [13, 14] implementing this policy measure is the next step for Australia to achieve tobacco endgame targets.

Yet, these policies pose challenges for retailers. While public support for retail reduction is strong, even among people who smoke, retailer groups have previously opposed such policies, citing anticipated revenue loss, competitive disadvantage relative to unlicensed sellers and insufficient transition support [15]. To date, research is yet to examine South Australian retailer perspectives on a phase‐out policy, as distinct from general retail restriction. Experiences from New Zealand, where legislated reduction in tobacco retailer numbers was repealed following government change, show both the potential and the fragility of ambitious public health measures [16].

Given this context, understanding retailers' perspectives on phasing out tobacco sales in South Australia is essential. Retailers are key actors in the tobacco supply chain and their ability to adapt will shape policy implementation, business continuity and public health outcomes. This study explored licensed tobacco retailer views in South Australia on managing the impacts of a potential tobacco retail sales phase out and forms of support sought for the transition.

## 2 Methods

### 2.1 Study Design

This study was designed as a single administration cross‐sectional survey. The Occupational Public Register of the South Australia Consumer and Business Services lists tobacco retail licence holders. A sample of licensed tobacco retailers in South Australia were contacted by email or phone and directed to a study website where they completed a confidential survey that asked how they anticipated tobacco retail phase out may affect their business. Unique contact details were publicly available for 621 of 1685 registered retail outlets, as common contacts were often shared by retail sites.

### 2.2 Measures

Participants were required to be aged 18 years or older, reside in South Australia and be employed by or affiliated with a licensed tobacco retailer in South Australia. The survey asked retailers which aspects of their retail practices they expect to be impacted by mandated retail phase out, what strategies they might implement to account for anticipated impacts, the support they would potentially seek to facilitate these strategies and time frames for implementation.

Closed survey items were rated from low (1) to high (10) importance or likelihood and open items captured free‐text responses. The survey took under 10 min to complete, was confidential and informed consent was provided when starting the survey. All survey items can be seen in Supporting Information 1. All respondents completing the survey received a $15 digital voucher for their time. The Flinders University Human Research Ethics Committee approved this study (#6650).

### 2.3 Analysis

Characteristics of respondents and item rating responses are reported in frequencies, means and standard deviations. Qualitative data were analysed in Excel to identify novel themes. Open‐text responses were analysed using an inductive thematic analysis approach. Codes were generated from the data, then grouped into themes and refined to consensus by two coders (EV, JT).

## 3 Results

This section describes respondent characteristics, then reports quantitative ratings of factors influencing decisions to cease tobacco sales and the support retailers would seek, before presenting qualitative findings on the impacts of a retail phase‐out.

### 3.1 Participants

A total of 70 respondents completed the survey (11% response rate). They were mostly employed at supermarkets (35%), convenience stores (22%), or other (e.g., food stores, caterers) (15%), with fewer petrol stations (9%), liquor stores (9%), licensed entertainment premises (6%) and tobacconists (3%) (five did not respond to this item). Respondents were more often business owners (75%) or managers (22%) than store attendants (3%) (five did not answer this item). All respondents sold tobacco, largely cigarettes (82%), loose tobacco (71%) and cigars (30%).

### 3.2 Support Needs and Response Patterns

When asked ‘To what extent do you believe the following factors will influence your decision to voluntarily cease the sale of tobacco products in the future?’ (Table 1), respondents identified legal, demand and profitability requirements as the most important factors and were less concerned about industry behaviour, media coverage and public health and wellbeing. This finding is consistent with the relatively low reported amount of pressure from sales representatives to stock tobacco products (M ± SD = 3.7 ± 3.3, *n* = 22).

**TABLE 1:** Relative importance of factors on voluntary tobacco retail phase out (*N* = 70).

Factor/response	M ± SD	*n*	%
*Influences on decision to cease tobacco sales*			
1. Increasing legal requirements designed to slow tobacco sales	7.7 ± 2.9	43	61.4%
2. The costs of selling tobacco products	7.4 ± 3.0	44	62.9%
3. Lack of profitability of tobacco	7.3 ± 2.7	48	68.6%
4. Decline in demand/reduced sales	7.3 ± 3.0	44	62.9%
5. Licence and insurance costs	6.7 ± 3.0	47	67.1%
6. Tobacco as a target for theft	6.7 ± 3.4	35	50.0%
7. Need for additional income from non‐tobacco products	6.3 ± 3.1	34	48.6%
8. Customer health and wellbeing	5.9 ± 2.9	32	45.7%
9. Community health and wellbeing	5.5 ± 3.1	34	48.6%
10. Actions of tobacco companies	4.8 ± 2.7	29	41.4%
11. Media coverage about tobacco and public health	4.5 ± 3.0	28	40.0%
12. Media coverage about Australia's retail sector	3.8 ± 2.5	29	41.4%
13. Behaviour of tobacco sales representatives	3.5 ± 2.3	28	40.0%
*Reason for requiring lead‐in time for ceasing tobacco sales*			
1. Seeking government support for business strategy	8.9 ± 1.5	23	32.9%
2. Seeking government financial support	8.6 ± 2.1	25	35.7%
3. Seeking industry support	8.0 ± 2.8	20	28.6%
4. Gradual phasing out of tobacco sales	7.8 ± 2.6	31	44.3%
5. Introducing new substitutionary products	7.0 ± 3.3	23	32.9%
6. Selecting a date to cease all tobacco product sales	6.6 ± 2.7	23	32.9%
7. Transfer or transition of employees	6.5 ± 3.4	18	25.7%
8. Nearby alternative retail locations (competitors)	5.1 ± 3.8	18	25.7%

*Note:* Relative importance was rated from 1 not at all, to 10 very much.

### 3.3 Support Needed

One in three retailers reported that they would need lead‐in time to transition away from tobacco sales (34.3%) and those responding identified securing external support for ceasing sales, both from government and industry, product substitution and the ability to cease sales gradually as key considerations. Retail competition and staffing were less important.

When asked ‘What would you do to account for impacts of upcoming legally enforced phase out of tobacco sales?’ (Table 2), respondents rated seeking general and financial support from both government and industry bodies as most important and considering product substitution as important. Converting businesses and location base changes were less strongly rated.

**TABLE 2:** Relative endorsement of potential responses to tobacco retail phase out (*N* = 70).

Factor/response	M ± SD	*n*	%
1. Seek government support for business strategy	8.2 ± 2.7	35	50.0%
2. Seek government financial support	8.1 ± 2.9	38	54.3%
3. Seek industry support for business strategy	7.2 ± 3.3	32	45.7%
4. Seek industry financial support	7.0 ± 3.6	35	50.0%
5. Introduce new substitutionary products	6.3 ± 3.3	27	38.6%
6. Convert the business to selling entirely different products	5.4 ± 3.8	22	31.4%
7. Stop tobacco sales at some business locations, and not others	5.0 ± 3.9	24	34.3%

*Note:* Relative importance was rated from 1 not at all to 10 very much.

Participants who provided an open text response (*n* = 38) to comment on phase‐out support strategies also described their views on the potential impacts of a retail tobacco sales phase‐out.

### 3.4 Risk and Declining Profitability of Illicit Trade

Retailers frequently linked declining profitability directly to the growth of the illicit tobacco trade, describing it as an economic burden that undermined the viability of continuing to stock tobacco products, rather than as a separate concern.

I don't like selling tobacco, but customers demand this product. In saying this, more than half our sales have now gone to illegal stores. (Supermarket, Owner)

Our small business has lost about $220,000 in sales [in] 2025 vs 2024, so that's about $175,000 in tax that the government would have received… If the government is serious about stopping smoking in Australia, make it illegal. (Supermarket, Owner)

We've seen a drop in our sales, as people are buying the cheaper illegal cigarettes. We cannot compete against their lower prices. We pay excise, [sales tax] and wages, not cash as they do to avoid these costs. (Licensed entertainment premises, Owner)

Beyond lost revenue, retailers also described the illicit trade as introducing personal and business safety risks, suggesting that for some, integrated financial and security concerns represented a tipping point towards supporting phase out.

[We'd need] protection from being extorted into selling tobacco products by organised crime syndicates. (Other retailer, Owner)

[There's] risk of being robbed. Although we hold only a few [products], we have noticed downturn in buying cigarettes. [So], we just have a small amount to offer customers something. (Liquor store, Owner)

### 3.5 Mixed Views on a Phase Out

Retailers were also asked about their views on three tobacco control measures that were legislated in New Zealand, including decreasing the number of tobacco retailers, reducing nicotine in tobacco and generational sales restriction. Reponses were mixed, with some retailers appreciating the role of such measures and others considering these to be over‐regulation.

Yes absolutely. We need to. (Convenience store, Owner)

I think all three are great ideas. (Other retailer, Owner)

[It's] over regulation. If you are going to ban them, do it but provide free health and support for several years prior. (Supermarket, Owner)

Happy for government to stop selling cigarettes in all shops and just have select few selling. So now [where] more are able to buy in the supermarket or bottle shops, [it's] just in specialised cig shops. (Liquor store, Owner)

Overall, participants' views on a tobacco retail phase out varied and they gave public health outcomes a lower priority than perceived impacts on business continuity.

## 4 Discussion

This exploratory survey of tobacco retailers in South Australia offers some insight into how businesses may respond to encouragement to cease tobacco sales voluntarily or through mandate. Overall, findings suggest that while some retailers view tobacco as increasingly unprofitable and vulnerable to competition from the illicit market. In terms of voluntarily ceasing tobacco sales, legal requirements, declining profits and reduced demands were key factors that would motivate a decision to stop selling tobacco. Around half indicated they would seek access to financial support from government or retailing organisations to transition their business to substitute products if tobacco retailing was phased out. These perspectives highlight opportunities and challenges for policymakers seeking to end tobacco retailing as part of Australia's tobacco endgame strategy [7, 14].

Our findings of factors that motivate consideration of ending tobacco sales are consistent with previous research from other Australian states [17, 18]. The persistence of illicit trade was repeatedly emphasised as a barrier to phase‐out compliance and as a factor that underpinned financial losses and security risks and perceptions of unfair competition. Similar concerns have been documented in other jurisdictions, such as New Zealand, where reversal of legislated retailer reductions highlighted the criticality of political support [16, 19]. Addressing illicit trade through stronger enforcement and support for businesses will therefore be crucial to achieving phase‐out policy implementation and maintaining confidence in the transition [12, 15]. Stricter regulation and reduced availability of tobacco are associated with lower uptake and greater quit success, [8, 9] and South Australia has the requisite licensing and enforcement measures to facilitate phase out.

In recent years, a large illicit tobacco market has grown in Australia with tobacco retailers being a primary distribution channel [20]. This has been accompanied by debate about tobacco taxation [21] and vaping product supply restrictions, [22] with some commentators, particularly the tobacco industry and other commercial actors, arguing for weakening of Australia's policies. However, given the large number of tobacco retailers throughout Australia that provide opportunity for organised crime to distribute products, reducing tobacco retailing should be part of the response to illicit tobacco trade [23]. Some retailers in our study appeared open to a tobacco retailing phase‐out due to the current situation of illicit trade. However, retailers may request financial and strategic support from government to enable this transition.

While some retailers were willing to consider exiting tobacco sales voluntarily, they noted a need for a transition period. This finding suggests that tobacco phase‐out policies may gain greater acceptance if implemented alongside well‐communicated support packages, clear timelines and opportunities for businesses to diversify away from tobacco. Experiences from New York City and Philadelphia, where licensing reforms (capping tobacco retailer licences) were accompanied by education and phased implementation, show that structured support can mitigate opposition and enhance compliance [11, 12].

These retailers emphasised economic and regulatory considerations as factors relevant to phasing out tobacco sales. Few retailers highlighted consumer or community health benefits as motivators for change. This finding underscores the disconnect between commercial interests and public health objectives, [2] and is consistent with the need to consider retailer business perspectives in framing tobacco control policies [24]. Tailoring communications to emphasise the declining profitability of tobacco, security risks and the potential for more lucrative substitute products may be more persuasive than health‐focused appeals.

This study has several limitations. The survey relied on voluntary responses from licensed retailers and while the sample included a mix of store types, tobacconists and entertainment venues were underrepresented. Additionally, while most respondents were owners or managers, some responses came from store attendants who may have less insight into business‐level decision‐making around tobacco sales. Self‐reported data are also subject to bias, although anonymity helps minimise this. Findings likely do not fully capture the diversity of retailer experiences in South Australia. Nonetheless, the survey provides initial evidence on South Australian tobacco retailer perspectives, an area for further research in tobacco control policy discourse [15].

## 5 Conclusion

This study adds further support to evidence highlighting that retailers already perceive tobacco as an economic liability, with sustained illicit trade and business uncertainty potentially increasing receptivity to a retail phase out. To maximise retailer support, governments should consider pairing policy measures with strategic support for retailers and clear timelines for transition to ceasing tobacco sales. By addressing these factors, tobacco retail phase out policies could achieve both public health objectives and business continuity, accelerating progress towards a tobacco endgame.

## Author Contributions

Conceptualisation: Joshua Trigg. Data curation: Joshua Trigg. Analysis: Joshua Trigg and Ella Vallelonga. Writing – original draft: Joshua Trigg and Ella Vallelonga. Writing – review and editing: Ella Vallelonga, Joshua Trigg, Ashleigh Guillaumier, Heewon Kang, Jennifer Baldock, Janet Hoek, Coral Gartner and Chelsea Falso. All authors reviewed and confirmed the approval of the submitted manuscript.

## Funding

This study was funded by the Australian National Health and Medical Research Council (NHMRC) grant #GNT1198301, with J.T., A.G. and C.F. also supported by the NHMRC Medical Research Future Fund grant #2022422.

## Ethics Statement

The Flinders University Human Research Ethics Committee approved this study (#6650).

## Conflicts of Interest

Joshua Trigg is an Editorial Board member of HPJA and a co‐author of this article. To minimise bias, they were excluded from all editorial decision‐making related to the acceptance of this article for publication.

## Supporting information


**Supporting Information: 1.** Business continuity and adapting to commercial tobacco endgame in Australia: Retail stakeholder views on transition strategies.

## Data Availability

Data are available from authors upon reasonable request.
