# Supplementary material for: Tobacco Retailer Perspectives on Potential Phase Out of Tobacco Sales in South Australia: An Exploratory Survey
Source: Health Promot J Austr. 2026 Oct 15;38(1):e70257. doi: 10.1002/hpja.70257 (PMC13672824; doi:10.1002/hpja.70257)
Supplement: Supplementary file 1 [file HPJA-38-0-s001.docx]

**Supplement 1**

***Business continuity and adapting to commercial tobacco endgame in Australia: Retail stakeholder views on transition strategies***

**Instructions**

Researchers at Flinders University are asking South Australian retailers about their views on the sale of tobacco products from businesses that have a license for this. The aim is to create a summary of retailer perspectives on the sale of tobacco products, and to specifically ask about views on the phasing out of retail tobacco sales, from different types of South Australian retailers.

This confidential survey takes 15 minutes to complete, and there is the option to receive an overall summary of the research findings about the South Australian retail sector.

If you would like to participate, in recognition of your contribution and participation time, you will be provided with a digital $15.00 Coles-Myer voucher. This voucher will be emailed to you after completion of the survey.

**For more information**

**—** A detailed description of research aims, and our privacy commitment can be accessed here: *summary link*

**—** You can also contact the researchers at *contact email*

**Please respond to the survey questions below by selecting or writing your answer**

1. **How would you describe your business?** (select main type)
   a. **☐** Tobacconist
   b. **☐** Convenience store
   c. **☐** Newsagency
   d. **☐** Liquor store
   e. **☐** Petrol station
   f. **☐** Supermarket
   g. **☐** Online only retailer
   h. **☐** Licensed entertainment premise
   i. **☐** Other (specify): __________
2. **What is your current position in the business?**
   a. **☐** Owner
   b. ☐ Store manager
   c. ☐ Cashier
   d. ☐ Customer service representative
   e. ☐ Visual merchandiser
   f. ☐ Buyer
   g. ☐ Inventory control specialist
   h. ☐ Sales associate
   i. ☐ Salesperson
   j. ☐ Retail warehouse worker
   k. ☐ Attendant
   l. ☐ Loss prevention manager
   m. ☐ Advertising / marketing manager
   n. ☐ Service supervisor
   o. ☐ Stocker
   p. **☐** Other (specify): __________
3. **What is your business postcode?** __________
a. ☐ Prefer not to say
b. ☐ Other (specify): __________
4. **Is your business part of a franchise or corporate chain?**
   a. **☐** Yes (specify): __________
   b. **☐** No
   c. **☐** Don’t know
   d. **☐** Prefer not to say
5. **Does your business sell any of the following products? (select all that apply)**
   a. **☐** Tobacco cigarettes
   b. **☐** Pouch tobacco
   c. **☐** Cigars
   d. **☐** Electronic cigarettes
   e. **☐** Nicotine-free electronic cigarettes
   f. **☐** Sheesha/hookah
   g. **☐** None of the above
   h. **☐** Other (specify): __________

**Next, we want to know your views on the retail sale of tobacco (please note that this is specific to combustible tobacco products i.e. cigarettes, pouch tobacco, cigars etc)**

6. **To what extent do you believe the following factors will influence your decision to voluntarily cease the sale of tobacco products in the future?** (please rate from 1 *not at all*, to 10 *very much*).

| **Not at all** | | |  |  |  |  | **Very much** | | |
| --- | --- | --- | --- | --- | --- | --- | --- | --- | --- |
| **1** | **2** | **3** | **4** | **5** | **6** | **7** | **8** | **9** | **10** |
| **The cost of selling tobacco products.** | | | | | | | | | |
| **☐** | **☐** | **☐** | **☐** | **☐** | **☐** | **☐** | **☐** | **☐** | **☐** |
| **Licence and insurance costs.** | | | | | | | | | |
| **☐** | **☐** | **☐** | **☐** | **☐** | **☐** | **☐** | **☐** | **☐** | **☐** |
| **Lack of profitability of tobacco.** | | | | | | | | | |
| **☐** | **☐** | **☐** | **☐** | **☐** | **☐** | **☐** | **☐** | **☐** | **☐** |
| **Decline in demand (reduced sales).** | | | | | | | | | |
| **☐** | **☐** | **☐** | **☐** | **☐** | **☐** | **☐** | **☐** | **☐** | **☐** |
| **The need for additional income from a diverse range of non-tobacco products.** | | | | | | | | | |
| **☐** | **☐** | **☐** | **☐** | **☐** | **☐** | **☐** | **☐** | **☐** | **☐** |
| **Tobacco as a target for theft.** | | | | | | | | | |
| **☐** | **☐** | **☐** | **☐** | **☐** | **☐** | **☐** | **☐** | **☐** | **☐** |
| **Community health and wellbeing.** | | | | | | | | | |
| **☐** | **☐** | **☐** | **☐** | **☐** | **☐** | **☐** | **☐** | **☐** | **☐** |
| **Customer health and wellbeing.** | | | | | | | | | |
| **☐** | **☐** | **☐** | **☐** | **☐** | **☐** | **☐** | **☐** | **☐** | **☐** |
| **Actions of tobacco companies.** | | | | | | | | | |
| **☐** | **☐** | **☐** | **☐** | **☐** | **☐** | **☐** | **☐** | **☐** | **☐** |
| **Behaviour of tobacco sales representatives.** | | | | | | | | | |
| **☐** | **☐** | **☐** | **☐** | **☐** | **☐** | **☐** | **☐** | **☐** | **☐** |
| **Media coverage about tobacco and public health.** | | | | | | | | | |
| **☐** | **☐** | **☐** | **☐** | **☐** | **☐** | **☐** | **☐** | **☐** | **☐** |
| **Media coverage about the Australian retail sector.** | | | | | | | | | |
| **☐** | **☐** | **☐** | **☐** | **☐** | **☐** | **☐** | **☐** | **☐** | **☐** |
| **Increasing legal requirements to slow tobacco sales.** | | | | | | | | | |
| **☐** | **☐** | **☐** | **☐** | **☐** | **☐** | **☐** | **☐** | **☐** | **☐** |

7. **Are there any other reasons important to you that would lead to your business to voluntarily stop selling tobacco products?** (please briefly describe).

8. **If a law was passed that required your business to cease tobacco sales by a set date, what would you do to account for the likely impacts?** (please rate from 1 *not at all*, to 10 *very much*).

| **Not at all** | | |  |  |  |  | **Very likely** | | |
| --- | --- | --- | --- | --- | --- | --- | --- | --- | --- |
| **1** | **2** | **3** | **4** | **5** | **6** | **7** | **8** | **9** | **10** |
| **Introduce new substitutionary products.** | | | | | | | | | |
| **☐** | **☐** | **☐** | **☐** | **☐** | **☐** | **☐** | **☐** | **☐** | **☐** |
| **Seek government financial support.** | | | | | | | | | |
| **☐** | **☐** | **☐** | **☐** | **☐** | **☐** | **☐** | **☐** | **☐** | **☐** |
| **Seek industry financial support.** | | | | | | | | | |
| **☐** | **☐** | **☐** | **☐** | **☐** | **☐** | **☐** | **☐** | **☐** | **☐** |
| **Seek government financial support for business strategy.** | | | | | | | | | |
| **☐** | **☐** | **☐** | **☐** | **☐** | **☐** | **☐** | **☐** | **☐** | **☐** |
| **Seek industry support for business strategy.** | | | | | | | | | |
| **☐** | **☐** | **☐** | **☐** | **☐** | **☐** | **☐** | **☐** | **☐** | **☐** |
| **Stop tobacco sales at some business locations, and not others.** | | | | | | | | | |
| **☐** | **☐** | **☐** | **☐** | **☐** | **☐** | **☐** | **☐** | **☐** | **☐** |
| **Convert the business to selling entirely different products.** | | | | | | | | | |
| **☐** | **☐** | **☐** | **☐** | **☐** | **☐** | **☐** | **☐** | **☐** | **☐** |

9. **What other key supports would be needed for your business to voluntarily stop selling tobacco products?** (please briefly describe).

10. **If the business you operate or work at decided to stop selling tobacco, how much lead-in time would it need to make this change successfully?**

| **This change would take:** | | | | | | | | | |
| --- | --- | --- | --- | --- | --- | --- | --- | --- | --- |
| ______Years |  | ______Months |  |  |  |  |  |  |  |

11. **What is the main reason for this amount of lead-in time?** (please rate 1 *not at all*, to 10 *very much*).

| **Not at all** | | |  |  |  |  | **Very likely** | | |
| --- | --- | --- | --- | --- | --- | --- | --- | --- | --- |
| **1** | **2** | **3** | **4** | **5** | **6** | **7** | **8** | **9** | **10** |
| **Select a date to cease all tobacco product sales.** | | | | | | | | | |
| **☐** | **☐** | **☐** | **☐** | **☐** | **☐** | **☐** | **☐** | **☐** | **☐** |
| **Gradual phase out of tobacco sales** | | | | | | | | | |
| **☐** | **☐** | **☐** | **☐** | **☐** | **☐** | **☐** | **☐** | **☐** | **☐** |
| **Introduce new substitutionary products** | | | | | | | | | |
| **☐** | **☐** | **☐** | **☐** | **☐** | **☐** | **☐** | **☐** | **☐** | **☐** |
| **Transfer and/or transition employees** | | | | | | | | | |
| **☐** | **☐** | **☐** | **☐** | **☐** | **☐** | **☐** | **☐** | **☐** | **☐** |
| **Close alternative retail locations** | | | | | | | | | |
| **☐** | **☐** | **☐** | **☐** | **☐** | **☐** | **☐** | **☐** | **☐** | **☐** |
| **Seek government financial support** | | | | | | | | | |
| **☐** | **☐** | **☐** | **☐** | **☐** | **☐** | **☐** | **☐** | **☐** | **☐** |
| **See industry support** | | | | | | | | | |
| **☐** | **☐** | **☐** | **☐** | **☐** | **☐** | **☐** | **☐** | **☐** | **☐** |
| **Seek government financial support for business strategy** | | | | | | | | | |
| **☐** | **☐** | **☐** | **☐** | **☐** | **☐** | **☐** | **☐** | **☐** | **☐** |

12. **Are there any other factors that would contribute to the amount of lead-in time mentioned above?** (please briefly describe).

13. **How much pressure is there from tobacco company sales representatives to continue stocking tobacco?** (please rate from 1 *no pressure*, to 10 *a lot of pressure*).

| **Amount of pressure.** | | | | | | | | | |
| --- | --- | --- | --- | --- | --- | --- | --- | --- | --- |
| **1 ☐** | **2 ☐** | **3 ☐** | **4 ☐** | **5 ☐** | **6 ☐** | **7 ☐** | **8 ☐** | **9 ☐** | **10 ☐** |

14. **Are there any strategies that your business currently uses to adapt to current government measures intended to reduce consumer tobacco purchases?** (please briefly describe).

**Last, we want to know your perspective on *how* tobacco sales restrictions could look.**

15. **If your business planned to stop selling tobacco (on its own), how likely would it be to use each of the following strategies for the transition?** (please rate from 1 *not at all likely*, to 10 *very likely*).

| **Not at all likely** | | |  |  |  |  | **Very likely** | | |
| --- | --- | --- | --- | --- | --- | --- | --- | --- | --- |
| **1** | **2** | **3** | **4** | **5** | **6** | **7** | **8** | **9** | **10** |
| **Pick a date to stop all tobacco product sales.** | | | | | | | | | |
| **☐** | **☐** | **☐** | **☐** | **☐** | **☐** | **☐** | **☐** | **☐** | **☐** |
| **Phase out tobacco product sales in stages.** | | | | | | | | | |
| **☐** | **☐** | **☐** | **☐** | **☐** | **☐** | **☐** | **☐** | **☐** | **☐** |
| **Seek government financial support.** | | | | | | | | | |
| **☐** | **☐** | **☐** | **☐** | **☐** | **☐** | **☐** | **☐** | **☐** | **☐** |
| **Seek industry financial support.** | | | | | | | | | |
| **☐** | **☐** | **☐** | **☐** | **☐** | **☐** | **☐** | **☐** | **☐** | **☐** |
| **Seek government support for business strategy.** | | | | | | | | | |
| **☐** | **☐** | **☐** | **☐** | **☐** | **☐** | **☐** | **☐** | **☐** | **☐** |
| **Seek industry support for business strategy.** | | | | | | | | | |
| **☐** | **☐** | **☐** | **☐** | **☐** | **☐** | **☐** | **☐** | **☐** | **☐** |
| **Stop tobacco sales at some business locations, and not others.** | | | | | | | | | |
| **☐** | **☐** | **☐** | **☐** | **☐** | **☐** | **☐** | **☐** | **☐** | **☐** |

16. **Are there any other strategies that your business would use to stop selling tobacco products?** (please briefly describe).

17. **In 2022, New Zealand passed three changes to their legislation on tobacco:**

- Decrease the number of retailers selling smoked tobacco products
- Reducing the amount of nicotine in smoked tobacco products
- Prohibiting the sale of smoked tobacco products to anyone born after 1 January 2009

**What are your thoughts on these amendments?** (please briefly describe).

18. **In 2023, New Zealand’s new government decided to repeal the changes mentioned above, and they will no longer be implemented. What are your thoughts on this decision?** (please briefly describe).

19. **Do you have any other additional thoughts or perspectives that you would like to share?** (please briefly describe).

20. **Would you be willing to be contacted at a later date to briefly and confidentially discuss your views on tobacco sales for a future study?**

| **YES ☐** | **NO ☐** |  |  |  |  |  |  |  |  |
| --- | --- | --- | --- | --- | --- | --- | --- | --- | --- |
|  | If selecting yes, please provide contact details here:**______________________** | | | | | | | | |
